# Incidence of and Risk Factors for SARS-CoV-2 Infection Among Vaccinated Healthcare Workers During Emergence of SARS-CoV-2 Gamma Variant in the Amazon Region, Brazil, 2021

**DOI:** 10.1093/cid/ciaf339

**Published:** 2025-07-30

**Authors:** Gemma Parra, Fernanda C Lessa, Evelyn Campelo, Tatyana C Amorim Ramos, Antonio R Vieira, Pritesh Lalwani, Lucia Y I Nichiata, Luciana Silva-Flannery, Charlene Siza, Kassia Janara Veras Lima, Aida Cristina Tapajos, Ariana Vieira, Mateusz Plucinski, Barbara J Marston, Juliette Morgan, Roberto J F Esteves, Cristiano Fernandes da Costa, Felipe G Naveca, Guilherme Araujo, Maria Clara Padoveze, for the Brazil Healthcare Personnel COVID-19 Team, Kiriath Rebello, Tirza Mattos, Andresa Rocha, Aguyda Barbosa, Lucas Thiago Pereira da Silva, Juliana F Silva, Kelly M Hatfield

**Affiliations:** Division of Healthcare Quality Promotion, Centers for Disease Control and Prevention, Atlanta, Georgia, USA; Division of Healthcare Quality Promotion, Centers for Disease Control and Prevention, Atlanta, Georgia, USA; COVID-19 Response—International Task Force, Centers for Diseases Control and Prevention, Atlanta, Georgia, USA; Fundação de Vigilância em Saúde do Estado do Amazonas, Manaus, Amazonas, Brazil; Fundação de Vigilância em Saúde do Estado do Amazonas, Manaus, Amazonas, Brazil; COVID-19 Response—International Task Force, Centers for Diseases Control and Prevention, Atlanta, Georgia, USA; Instituto Leônidas e Maria Deane (ILMD), Fiocruz Amazônia, Manaus, Amazonas, Brazil; Laboratory of Infectious Diseases and Immunology, ILMD/Fiocruz Amazônia and PPGIBA/ICB-UFAM, Manaus, Amazonas, Brazil; Department of Collective Health Nursing, Universidade de São Paulo, Escola de Enfermagem, São Paulo, São Paulo, Brazil; COVID-19 Response—International Task Force, Centers for Diseases Control and Prevention, Atlanta, Georgia, USA; Division of Healthcare Quality Promotion, Centers for Disease Control and Prevention, Atlanta, Georgia, USA; COVID-19 Response—International Task Force, Centers for Diseases Control and Prevention, Atlanta, Georgia, USA; Hospital Pronto Socorro 28 de Agosto, Manaus, Amazonas, Brazil; Hospital Pronto Socorro Platão Araújo, Manaus, Amazonas, Brazil; Hospital Pronto Socorro Platão Araújo, Manaus, Amazonas, Brazil; COVID-19 Response—International Task Force, Centers for Diseases Control and Prevention, Atlanta, Georgia, USA; COVID-19 Response—International Task Force, Centers for Diseases Control and Prevention, Atlanta, Georgia, USA; South America Regional Office, Centers for Diseases Control and Prevention, Brasília, Federal District, Brazil; South America Regional Office, Centers for Diseases Control and Prevention, Brasília, Federal District, Brazil; Conselho de Secretários Municipais de Saúde do Amazonas, Manaus, Amazonas, Brazil; Instituto Leônidas e Maria Deane (ILMD), Fiocruz Amazônia, Manaus, Amazonas, Brazil; Laboratório Central do Amazonas (LACEN), Manaus, Amazonas, Brazil; Department of Collective Health Nursing, Universidade de São Paulo, Escola de Enfermagem, São Paulo, São Paulo, Brazil

**Keywords:** SARS-CoV-2, COVID-19, healthcare workers, vaccination, variants of concern

## Abstract

**Background:**

The emergence of new severe acute respiratory syndrome coronavirus 2 (SARS-CoV-2) variants poses a significant threat to public health and healthcare workers (HCWs). The SARS-CoV-2 Gamma variant emerged in Manaus, Brazil, in December 2020, leading to a large wave of infections and deaths.

**Methods:**

We conducted a prospective cohort study among HCWs at 2 hospitals in Manaus from 31 March to 31 May 2021. HCWs were followed up for 3 weeks with nasal swabs collected at baseline and weekly (total: 4 visits), regardless of symptoms, for SARS-CoV-2 testing. Blood samples were collected for SARS-CoV-2 serology at baseline. Staff collected data on symptoms, healthcare, and community exposures, and vaccination status at each visit. We evaluated coronavirus disease 2019 (COVID-19) attack rate, risk factors for disease, and differences in rates by vaccination status.

**Results:**

We included 771 HCWs (388 and 383 per hospital). The median age was 40 years (IQR: 31–48), 600 (77.8%) were female, 449 (58.2%) were nurses, and 568 (73.6%) were fully vaccinated at enrollment. We identified 16 SARS-CoV-2 incident infections for an overall attack rate of 5.2 per 1000 HCW-weeks. Of the 16 infected HCWs, 12 were fully vaccinated (attack rate: 5.2/1000 HCW-weeks vs 5.9/1000 HCW-weeks among unvaccinated). All 12 vaccinated HCWs tested negative after 1-week follow-up, while 2 of the 3 infected unvaccinated individuals remained positive at follow-up (*P* < .05).

**Conclusions:**

The COVID-19 attack rate among this cohort of HCWs with high vaccine coverage was low. Among vaccinated HCWs who tested positive, none tested positive after 1 week, indicating more rapid viral clearance vs unvaccinated HCWs. HCW vaccination is an important measure for HCW safety and more resilient healthcare systems.

## Teaser

This study investigated risk factors for SARS-CoV-2 infection among a cohort of healthcare workers in 2 hospitals in Manaus, Brazil. Data were collected after a large wave of infections and deaths, coinciding with the emergence of the Gamma (P1) variant.

The severe acute respiratory syndrome coronavirus 2 (SARS-CoV-2) Gamma (P.1) variant of concern emerged in the Amazonas state of Brazil in late December 2020 and resulted in a second wave of coronavirus disease 2019 (COVID-19) infections and deaths [1–4]. This second wave, approximately 7 months after the initial wave, was characterized by substantial mortality, collapse in the healthcare system, and socioeconomic impact. Studies estimated that, during the first wave, 29% to 45% of the population in the state's capital of Manaus was infected [5–7]. Despite this high estimated rate of infection during the first COVID-19 wave, Manaus reported a 105% increase in COVID-19 hospitalizations from 1 to 15 January 2021, with hospitalizations increasing from 1905 to 3905 [8], leading to concerns about the Gamma variant's increased transmissibility, severity, and potential for reinfection (antibody escape), which was later shown in several studies [2, 9–12].

Healthcare workers (HCWs) play a critical role in mitigating the COVID-19 pandemic and have experienced significant impacts [13, 14]. Modeling data from South Africa, Kenya, Eswatini, and Colombia show the substantial societal costs incurred due to SARS-CoV-2 infections among HCWs, particularly in countries where they face disproportionately higher infection rates compared with the general population [15]. Studies conducted during the initial COVID-19 wave in Brazil revealed that nearly 43% of symptomatic HCWs tested positive for SARS-CoV-2 by molecular testing, with evidence suggesting many experienced asymptomatic infections based on serological data [16, 17]. A World Health Organization (WHO) report estimated that over 115 500 HCWs worldwide may have died from COVID-19 between January 2020 and May 2021, likely an underestimate due to underreporting in many countries [18]. The significant loss of lives takes a heavy toll on our global healthcare workforce, which comprises 135 million individuals distributed unevenly across nations [19]. Ensuring the protection of our healthcare workforce is crucial to sustain the delivery of healthcare to the population.

On 17 January 2021, Brazil received 6 million doses of CoronaVac, an inactivated whole-virus vaccine, initially imported from Sinovac, China, later produced locally by Butantan Institute, São Paulo, Brazil [20, 21]. This was followed on 22 January 2021 by 2 million doses of ChAdOx1 nCoV-19 vaccine (AZD1222; Oxford/AstraZeneca) imported from the Serum Institute in India. Since March 2021, Fiocruz in Brazil has produced the ChAdOx1 nCoV-19 vaccine locally [22–24]. CoronaVac is a whole inactivated virus vaccine, requires 2 doses, and has an efficacy of 50.7% (95% CI: 36.7%–81.52%) against symptomatic COVID-19 [25]. ChAdOx1 delivers a chimpanzee adenovirus encoding the SARS-CoV-2 spike glycoprotein and requires 2 doses [26]. Phase 3 clinical trials in Brazil, South Africa, and the United Kingdom found an efficacy of 55.1% against symptomatic COVID-19 when doses were administered less than 6 weeks apart, increasing to 81.3% when doses were 12 weeks apart or longer [27, 28].

Healthcare workers were prioritized for vaccination by the Brazilian Ministry of Health, and a vaccination campaign among HCWs in the state of Amazonas started on 19 January 2021 [29]. By 31 March 2021, the state achieved 52.9% coverage (51 000 doses) for the first dose and 84% coverage (43 000 doses) for the second dose among those who received the first dose [8]. We conducted an investigation to evaluate COVID-19 attack rate and characterize risk factors for infection and differences in attack rate and viral shedding by vaccination status among HCWs from 2 large public hospitals in Manaus from 31 March to 31 May 2021.

## METHODS

### Study Setting

Manaus, the capital of Amazonas state, is the most populous city in the Brazilian Amazon region, estimated to have 2.06 million inhabitants [30]. From 1 January–31 March 2021, Manaus reported 77 967 new confirmed COVID-19 cases and 8609 COVID-19–related hospitalizations [31]. We enrolled HCWs from 2 major public hospitals in Manaus: (1) Hospital Pronto Socorro 28 de Agosto (HPS 28), a 379-bed hospital for adult and pediatric patients, including 64 COVID-19 intensive care unit (ICU) beds, located in the south-central region of Manaus and with approximately 2800 HCWs, and (2) Hospital Pronto Socorro Platão Araújo (HPS Platão), a 189-bed hospital for adult patients only, including 33 ICU beds and 22 step-down unit beds, located in the eastern region of Manaus and with approximately 1900 HCWs. Throughout January–March 2021, these hospitals were dedicated to COVID-19 admissions, suspending other services. Despite having established infection prevention and control (IPC) teams, both hospitals faced overcapacity, necessitating patient airlifting to other states for care.

### Study Design and Population

We conducted a prospective, observational cohort study of HCWs from diverse cadres. Healthcare workers from both hospitals were enrolled from 31 March–31 May 2021, and were followed up for 3 weeks, for a total of 4 visits. A nasal swab was collected at enrollment and weekly regardless of symptoms, and a blood specimen was obtained at enrollment. Healthcare workers were eligible for inclusion if they had direct contact with patients or were in contact with patients' surroundings (eg, hospital cleaners), were 18 years of age or older, and had a way to be contacted by phone. To ensure consistent exposure to the hospital environment, we enrolled only HCWs who reported working at least 20 hours per week. Healthcare workers were excluded if they were an employee of the hospital but not on active duty since December 2020, worked fewer than 20 hours per week at the study hospital, or refused collection of nasal swab or blood sample at enrollment.

### Data Collection and Definitions

Study staff were trained on specimen and data collection and recruited HCWs at the hospital 5 days per week, spanning multiple shifts to accommodate HCWs across all work schedules. Data were collected using a standardized tool and included demographics, occupation, chronic conditions, occupation risks, COVID-19 community exposures, COVID-19 vaccination, history of COVID-19, and clinical symptoms. Weekly follow-ups included a shorter questionnaire on exposures, clinical symptoms, personal protective equipment (PPE) use, and COVID-19 vaccination updates. Documentation of COVID-19 vaccination status, including formulation, dose, and dates, was obtained from participant immunization cards or the state COVID-19 immunization registry [32].

We collected data on 12 aerosol-generating procedures (AGPs), categorizing them by risk level based on WHO guidelines (Supplementary Table 1). We assessed HCWs’ involvement in performing, assisting, or being present during AGPs. Vaccination status was classified as fully vaccinated (2 doses ≥14 days before specimen collection), partially vaccinated (1 dose ≥14 days before specimen collection), or unvaccinated (no documented doses). Infection despite vaccination was defined as confirmed SARS-CoV-2 infection by real-time reverse-transcriptase polymerase chain reaction (RT-PCR) in fully vaccinated HCWs. Prior COVID-19 infection relied on self-reporting by HCWs.

### Laboratory Methods

Self-collected nasal swabs were placed in viral transport medium, stored at 2°C–4°C, and transported in coolers within 48 hours after collection to Laboratório Central do Amazonas (LACEN-AM) for real-time RT-PCR for SARS-CoV-2 using AllplexTM 2019-nCoV Assay [33]. The RT-PCR results were expressed as the cycle threshold (Ct) for the gene encoding the nucleocapsid protein (N gene), as previously described [34]. All samples with sufficiently low Ct values (Ct <30) from RT-PCR testing were defined as a case and underwent whole-genome sequencing using a COVIDSeq library preparation kit (Illumina) at Fiocruz-Amazonas, as previously described [2]. The Ct values from the qualitative PCR were used as a proxy measure for viral load.

Whole blood (4 mL) was placed in EDTA-coated tubes and plasma was tested using previously validated in-house enzyme-linked immunosorbent assay (ELISA) for SARS-CoV-2 nucleocapsid (NC) immunoglobulin G (IgG) antibody titers (residues 1–410; GenBank: QHD43432.2) [7], and commercially available ELISA for SARS-CoV-2 S1 IgG titers (EUROIMMUN Anti-SARS-CoV-2 S1 Curve ELISA) [35]. An anti–SARS-CoV-2 NC IgG antibody reactivity index (RI) was expressed as the ratio between optical density of the patient sample and the negative control. All samples with an RI of 1.5 or greater were considered positive. For anti–SARS-CoV-2 S1 IgG, we used the manufacturer’s cutoff of more than 35.2 binding antibody units/mL (BAU/mL) to determine reactivity. Reactive samples were further classified as low-titer if BAU/mL was 35.3–260.03 and high-titer if greater than 260.03 based on the distribution of the serologic titers among our HCW cohort. This classification is consistent with data from ChAdOx1 nCoV-19 clinical trial that found a titer of 264 BAU/mL or greater to be correlated with protection against SARS-CoV-2 infection [36].

### Statistical Analyses

We summarized HCW characteristics by hospital using frequencies for categorical variables and medians with ranges for continuous variables. Attack rates were calculated as the number of new infections per 1000 susceptible HCW-weeks. Susceptibility to infection was defined as testing negative for SARS CoV-2 at enrollment or in previous study visits. A Fisher's exact test was conducted to assess the relationship between vaccination and persistent positive tests. Logistic regression with generalized estimating equations (GEEs) was used to calculate odds ratios (ORs) across exposures for our primary outcome of positive RT-PCR–confirmed SARS-CoV-2 infection. This nested model accounts for clustering at the facility level and for repeated measures for each HCW across the study period [37].

All analyses were performed using SAS version 9.4 (SAS Institute, Cary, NC). This project was reviewed and approved by the Institutional Review Board from Universidade de São Paulo and the Brazilian National Ethics Committee. This activity was reviewed by the Centers for Disease Control and Prevention (CDC), deemed non-research, public health investigation, and was conducted consistent with applicable federal law and CDC policy. Written informed consent was obtained from all participants prior to enrollment.

## RESULTS

We included 771 HCWs, 383 from HPS 28 and 388 from HPS Platão. The median age was 40 years (IQR: 31–48 years), 600 (78%) were female, 449 (58%) were either a registered nurse or nurse assistant, and 660 (86%) reported no comorbidities. Of the 466 HCWs who reported COVID-19 prior to study enrollment, 84 (18%) reported onset within 90 days of recruitment. COVID-19 vaccination coverage for at least 1 dose was 84% (644/771), with the majority receiving CoronaVac (98%; 628/644) (Table 1). Of the 771 HCWs, 38% reported caring for patients with suspected or confirmed COVID-19 in the 2 weeks prior to study enrollment or thereafter. Among those, 91% reported wearing a cloth mask most of the time or always, 383 (34%) reported wearing medical masks, and 247 (22%) reported wearing N95 respirators (Supplementary Figure 1). Overall, 70% of HCWs did not report exposure to potential AGPs, while 18% encountered high-risk exposures, 8% medium-risk, and 4% low-risk exposures during the study (Supplementary Table 2).

**Table 1.** Demographic Characteristics of Healthcare Workers in 2 Hospitals in Manaus, Brazil—March–May 2021

	HPS Platão (n = 388)	HPS 28 (n = 383)	Total, No. (%) (N = 771)
Age, median [IQR], y	41 [32–47]	40 [30–48]	40 [31–48]
Sex			
Male	67	104	171 (22.18)
Female	321	279	600 (77.82)
Occupation			
Nurse assistant	198	133	331 (42.9)
Registered nurse	66	52	118 (15.3)
Security	10	59	69 (8.9)
Environmental cleaning staff	30	36	66 (8.6)
Administrative staff	12	15	27 (3.5)
Physical therapist	12	21	33 (4.28)
Physician	8	16	24 (3.1)
Other	52	51	118 (15.3)
No. of healthcare facilities working at			
1	289	303	592 (76.8)
2	96	78	174 (22.6)
3	3	2	5 (0.65)
No. of hours with patient face-to-face contact during typical work week (1–80 hours), median [IQR]	20 [10–30]	30 [1–40]	25 [10–39]
HCW regularly provides hands-on medical care			
Yes	241	184	425 (55.1)
No	147	199	346 (44.9)
Smoking status			
Every day	11	20	31 (4)
Somedays	16	21	37 (4.8)
Not at all	361	342	703 (91.2)
Participated in physical activity in the last 30 d			
Yes	132	146	278 (36.1)
No	256	237	493 (63.9)
Comorbidities			
Yes	56	55	111 (14.4)
No	332	328	660 (85.6)
Baseline serology^a^			
Nonreactive	13	15	28 (3.6)
Reactive low titer	181	191	372 (48.3)
Reactive high titer	194	177	371 (48.1)
SARS-CoV-2 infection prior to study enrollment			
Yes—≥90 d previously	149	196	345 (45)
Yes—within the previous 90 d	50	34	84 (11)
Yes—did not report date of infection	22	15	37 (4.8)
No	167	138	305 (40)
Vaccine type among those fully or partially vaccinated at study enrollment			
CoronaVac	313	315	628 (80.1)
AstraZeneca	9	7	16 (2.08)
Unvaccinated	66	61	127 (17.3)

Abbreviations: BAU, binding antibody units; HCW, healthcare worker; HPS Platão, Hospital Pronto Socorro Platão Araújo; HPS 28, Hospital Pronto Socorro 28 de Agosto; IQR, interquartile range; SARS-CoV-2, severe acute respiratory syndrome coronavirus 2.

^a^Serology levels (BAU/mL) were defined as non-reactive if <35.2, reactive low titer if 35.2–260.03, and reactive high titer if >260.03.

**Figure 1.**
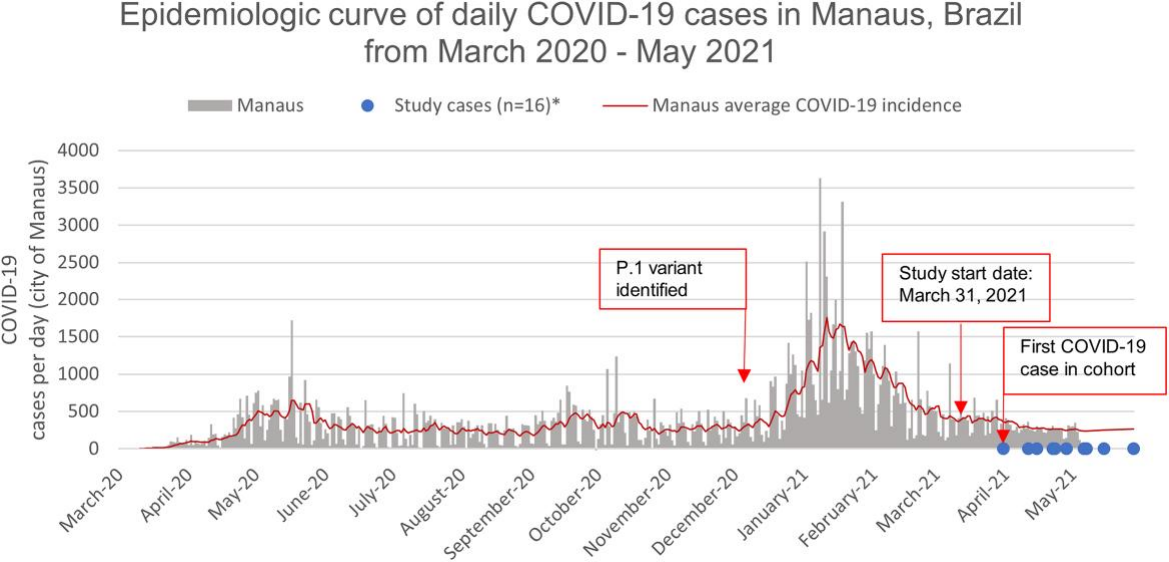
Epidemiologic curve of daily COVID-19 cases in Manaus, Brazil, from March 2020 to May 2021. Abbreviation: COVID-19, coronavirus disease 2019. *The blue dot may represent >1 study case. Laboratory-confirmed COVID-19 data was extracted from the Fundação de Vigilância em Saúde do Amazonas (FVS-AM) website. https://www.fvs.am.gov.br [8].

SARS-CoV-2 infection was confirmed in 16 (2%) participants during the follow-up period, and none were hospitalized. The first case occurred on 12 April 2021, during the later stages of the epidemic curve, at a point when daily cases of COVID-19 in Manaus were declining (Figure 1). The median age among infected HCWs was 43.5 years, with 6 (38%) reporting a prior infection. Most of the infected HCWs provided regular hands-on medical care (9 HCWs). Among those, 4 reported providing direct care for a patient with suspected or confirmed COVID-19. Of those 4 HCWs, 1 reported no PPE use in the week before testing positive and the remaining 3 reported only using a cloth mask with no other PPE.

**Figure 2.**
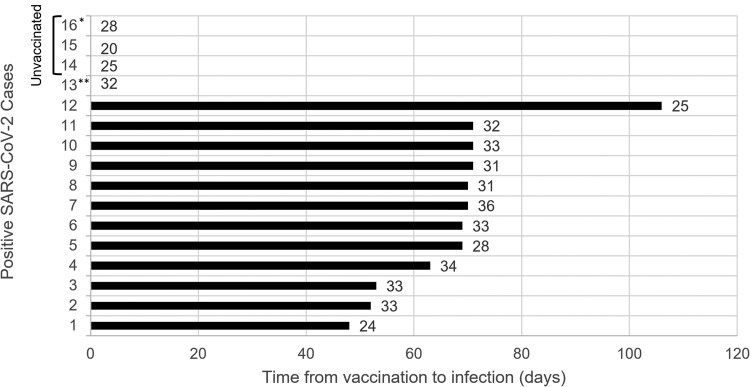
SARS-CoV-2 Ct values for infected HCWs (n = 16) and time to infection after full vaccination (2 vaccine doses >14 days prior to positive specimen) (n = 12)—Manaus, Brazil, March–May 2021. Unvaccinated HCWs are displayed on the *y*-axis time point 0 days since they were unvaccinated. *Had persistent viral shedding (defined as testing positive for >1 weekly visit). **Partially vaccinated (1 dose). Abbreviations: Ct, cycle threshold; HCW, healthcare worker; SARS-CoV-2, severe acute respiratory syndrome coronavirus 2.

The majority of infected HCWs (12/16; 75%) were fully vaccinated, while 3 infections occurred among unvaccinated HCWs and 1 among a partially vaccinated HCW. COVID-19 symptoms were reported by 58% of vaccinated HCWs compared with 75% of unvaccinated or partially vaccinated HCWs. All 3 HCWs who reported fever were unvaccinated. The median time from completion of the 2-dose vaccine schedule to infection among the 12 infections in vaccinated HCWs was 68.5 days (range: 48–106 days). The median Ct among these cases was 32.5 (range: 24–36) compared with 26.5 (range: 20–32) among unvaccinated HCWs (Figure 2). Two out of the 3 unvaccinated HCWs remained positive after 1 week, while none of the infected vaccinated HCWs tested positive on the 1-week follow-up visit (*P* = .03).

The overall COVID-19 attack rate per 1000 susceptible HCW-weeks was 5.2: 5.9 for males and 5.0 for females. By job category, the attack rate was highest among environmental cleaning staff and nurse assistants (7.7 and 6.9 per 1000 HCW-weeks, respectively) (Table 2). The attack rate was higher for those aged 60 years and older compared with the younger age group (9.3 vs 5.1 per 1000 HCW-weeks) and for those without prior COVID-19 (7.5 per 1000 HCW-weeks). Healthcare workers with known direct contact with patients with confirmed or suspected COVID-19 had a relatively lower attack rate than HCWs who reported no direct contact with patients with COVID-19 (3.9 vs 6.5/1000 HCW-weeks). The attack rate among participants who received at least 1 dose of a COVID-19 vaccine was 5.1 per 1000 HCW-weeks compared with an attack rate of 5.9 per 1000 HCW-weeks among unvaccinated HCWs (Table 2). Vaccination was not statistically associated with a decreased risk of infection (OR: .89; 95% CI: .25–3.15). Healthcare workers with high-titer reactivity to the S1 or spike protein were estimated to have a 56% lower risk of SARS-CoV-2 infection, and those with low-titer reactivity had a 33% lower risk compared with those with nonreactive serology (Table 2). However, these findings were not statistically significant. Due to high Ct values, only 6 specimens (2 from unvaccinated and 4 from vaccinated HCWs) were sequenced, and all of them were identified as Gamma (P.1).

**Table 2.** Attack Rates and Risk Ratios Among Healthcare Workers (HCWs) Who Tested Negative for SARS-CoV-2 at Study Enrollment or Previous Visit (Susceptible HCWs), in 2 Hospitals in Manaus, Brazil—March–May 2021

	HCW-Weeks	New Cases	Attack Rate (per 1000 HCW-Weeks)	Odds Ratio	95% CI
Sex					
Male	676	4	5.92	Ref	…
Female	2385	12	5.03	.85	.28–2.63
Age					
<60 y	2953	15	5.08	Ref	…
≥60 y	108	1	9.26	1.82	.24–13.99
Occupation					
Nurse assistant (nurse technician)	1302	9	6.91	2.63	.57–12.21
Registered nurse	469	2	4.26	1.62	.23–11.53
Security officers	273	1	3.66	1.39	.13–15.38
Environmental cleaning staff	259	2	7.72	2.94	.41–20.99
Others	758	2	2.64	Ref	…
Number of healthcare facilities employed at					
1	2348	14	5.96	Ref	…
2	693	2	2.89	.48	.11–2.13
≥3	20	0	0	…	…
Comorbidities					
No	2627	11	4.19	Ref	…
Yes	434	5	11.5	2.77	.96–8.02
Household size					
<5	2649	15	5.66	Ref	…
≥5	412	1	2.43	.43	.6–3.24
Prior SARS-CoV-2 infection^a^					
No reported previous infection	1202	10	8.32	Ref	…
Previous infection	1859	6	3.22	.39	.14–1.06
Baseline serology^b^					
Nonreactive	109	1	9.17	Ref	…
Reactive low titer	1473	9	6.11	.67	.08–5.29
Reactive high titer	1479	6	4.06	.44	.05–3.67
Vaccination status					
Unvaccinated	511	3	5.87	Ref	…
Partially vaccinated	247	1	4.05	.69	.07–6.65
Fully vaccinated	2303	12	5.21	.89	.25–3.15
Hospital unit					
Non-ICU	2340	10	4.27	Ref	…
ICU	721	6	8.32	1.95	.71–5.40
Attack rates for weekly exposures					
Direct contact with a patient with suspected or confirmed COVID-19					
No	1841	12	6.52	Ref	…
Yes	1114	4	3.56	.55	.18–1.71
Exposure to a confirmed or suspected COVID-19 case outside of hospital					
No	2959	14	4.73	Ref	…
Yes	102	2	19.61	4.14	.94–18.76
Direct contact with patient environment					
No	1578	9	5.70	Ref	…
Yes	606	2	3.30	.58	.12–2.68
Highest procedure risk category that week					
No AGPs^c^	2184	12	5.49	Ref	…
Low-risk	42	1	23.81	4.33	.56–34.8
Medium-risk	191	2	10.47	1.92	.43–8.62
High-risk	644	1	1.55	.28	.04–2.17

Abbreviations: AGP, aerosol generating procedure; BAU, binding antibody units; COVID-19, coronavirus disease 2019; HCW, healthcare worker; ICU, intensive care unit; Ref, reference; SARS-CoV-2, severe acute respiratory syndrome coronavirus 2.

^a^Self-reported previous infection within 90 d.

^b^Serology levels (BAU/mL) for the S1 or spike protein were defined as nonreactive if <35.2, reactive low titer if 35.2–260.03, and reactive high titer if >260.03.

^c^High-risk: tracheal intubation, noninvasive ventilation, cardiopulmonary resuscitation, manual ventilation, bronchoscopy, sputum induction; medium-risk: nebulization and high-flow oxygen; low-risk: airway suctioning, extubation, respiratory physiotherapy, bronchoalveolar lavage.

## DISCUSSION

In this large prospective cohort of 771 HCWs with high vaccine coverage, we identified 16 incident SARS-CoV-2 infections, 12 of which were among fully vaccinated individuals, and approximately 50% of those having the infection within 2 months after being fully vaccinated with 2 doses. During the 3-week follow-up period, the overall attack rate was 5.1 per 1000 HCW-weeks: 5.2 for fully vaccinated HCWs and 5.9 for unvaccinated HCWs.

Environmental cleaning staff experienced the highest SARS-CoV-2 attack rate (7.7 per 1000 HCW-weeks); although not significant in our study, it mirrors findings from similar studies conducted in Brazil and the United States [38, 39]. This could be attributable to differences in training among these employees, who are often outsourced and have low wages with high turnover rates, which may lead to a lack of training aligned with IPC guidelines. Environmental cleaning staff may also have other exposures for increased transmission risk outside of the hospital because of lower socioeconomic status, such as taking public transportation, working at additional healthcare facilities, and household crowding. In contrast, HCWs who reported direct contact with a patient with suspected or confirmed COVID-19 had a lower risk of infection (OR: .55; 95% CI: .18–1.71) than those who did not, despite being directly involved in patient care. Healthcare workers in direct contact are not necessarily at a higher risk of infection, possibly due to their heightened awareness of transmission risk. Despite the nonsignificant result, training for all individuals working in hospital environments, regardless of direct patient contact, is important to mitigate potential exposures.

We did not find any significant association between COVID-19 and healthcare exposures in our study. There are many possible explanations to our lack of significant findings. The low attack rate in this cohort may limit the statistical power necessary to reveal any significant associations between exposure and risk of infection. The attack rate in our study is notably lower than what was observed in Manaus just a few months prior (76% in the general population in the month of October 2020 vs 2% among our cohort participants) [40], possibly due to the short follow-up period and high immunity from prior infection or vaccination at baseline. At enrollment, 74% were fully vaccinated and 61% self-reported prior SARS-CoV-2 infections. Baseline serology revealed that 96% of HCWs had reactive IgG titer values (>35.2 BAU/mL) to the S1 or spike protein, half of which were high (>260 BAU/mL), which could be due to either infection or vaccination. Additionally, enrollment for this study began approximately 3 months after the Gamma variant was first identified and 2 months after a large wave of SARS-CoV-2 infections that devastated the region (Figure 1). These data suggest that our HCW cohort had some immunity (through prior infection or vaccine) against SARS-CoV-2 infection at enrollment, likely protecting against the Gamma strain.

In this study, prior infection and baseline serology levels were stronger protective factors compared with vaccination, although none were statistically significant. This observation could be the result of several factors: (1) the type of vaccine (inactivated whole-virus vaccine—CoronaVac), which has an efficacy of 51% against symptomatic disease compared with more than 90% for molecular RNA vaccines used in the United States [40], and (2) interaction between serology, prior infection, and vaccination since those with prior infection and subsequent vaccination might exhibit a more robust immune response post-vaccination with high levels of antibodies in our study baseline. Although vaccination was not a statistically significant protective factor against COVID-19 infection, those who were vaccinated had no fever, presumptive low viral load estimated by the high Ct values, and rapid clearance, with the subsequent nasal swab at the 1-week follow-up being negative. These findings are similar to other cohort studies in other countries, which have also shown faster viral clearance time among vaccinated individuals compared with unvaccinated individuals [41, 42].

Our study has many strengths, notably a large cohort of HCWs in a highly affected COVID-19 region. The collection of respiratory samples for 3 weeks regardless of symptoms allowed for the identification of asymptomatic and mildly symptomatic individuals. Despite these strengths, the following limitations should be noted:

1. The use of PPE was only assessed among HCWs who reported caring for patients with COVID-19, which limited our ability to compare PPE use among all of those enrolled in the cohort.
2. The study was conducted at only 2 hospitals in Manaus and may not be generalizable to other regions in the country or in countries that used other vaccine formulations, such as molecular RNA vaccines. We also did not evaluate anyone who received a booster dose.
3. A large proportion of the HCWs enrolled had just completed 2 doses of COVID-19 vaccination or had been infected in the large wave 2 months prior, which likely increased population immunity, leading to low numbers of infected HCWs identified.
4. The study was limited by the short follow-up period of 3 weeks, which limits the ability to thoroughly evaluate the long-term impact of a vaccine in this population [43].

Lastly, variables such as prior infection and PPE use were self-reported and have the potential for recall bias. Despite these limitations, the study describes important HCW characteristics, including IPC practices, in the country in Latin America with the second-highest number of COVID-19–related deaths [44].

In conclusion, because HCWs are at high risk of infection during pandemics and epidemics of respiratory pathogens, cohort studies involving these individuals can provide valuable information on infection burden and risks. Protection of HCWs is critical to keep healthcare systems operating and delivering healthcare to the population. Our study corroborates the published literature demonstrating a low COVID-19 attack rate among a highly vaccinated population, with a nonsignificant suggestion of a more rapid viral clearance among those vaccinated. It also shows the need for enhanced IPC training across all cadres of HCWs, particularly among nonclinical staff such as environmental cleaners, coupled with adequate availability of PPE to avoid suboptimal prevention practices during patient care activities at the peak of a pandemic. By considering the lessons learned from this study and prioritizing surveillance, education, and vaccination, we can better equip ourselves to mitigate the impact of future outbreaks and safeguard public health.

## Supplementary Material

ciaf339_Supplementary_Data

## Supplementary Data

Supplementary materials are available at *Clinical Infectious Diseases* online. Consisting of data provided by the authors to benefit the reader, the posted materials are not copyedited and are the sole responsibility of the authors, so questions or comments should be addressed to the corresponding author.

## Notes

***Acknowledgments.*** The authors acknowledge the following members of the Brazil Healthcare Personnel COVID-19 team: Kiriath Rebello and Tirza Mattos, Lacen-AM; Andresa Rocha, Hospital Pronto Socorro Platão Araújo; Aguyda Barbosa, Instituto Leônida and Maria Deane, Fiocruz; Lucas Thiago Pereira da Silva, Escola de Enfermagem—Universidade de São Paulo; and Juliana F. Silva and Kelly M. Hatfield, Centers for Disease Control and Prevention. The authors also acknowledge the hospital administration for their support to allow the study to be performed in the hospital premises. The authors also thank all study staff and the hard-working and dedicated healthcare workforce from Hospital Pronto Socorro 28 de Agosto and Hospital Pronto Socorro Platão Araújo who, in addition to being the frontline of the coronavirus disease 2019 (COVID-19) response in Manaus, volunteered for this study.

***Data availability.*** The datasets generated and analyzed during the current study are available from the corresponding author on reasonable request.
