# Supplementary material for: Incidence of and Risk Factors for SARS-CoV-2 Infection Among Vaccinated Healthcare Workers During Emergence of SARS-CoV-2 Gamma Variant in the Amazon Region, Brazil, 2021
Source: Clin Infect Dis. 2025 Jul 30;81(3):451–8. doi: 10.1093/cid/ciaf339 (PMC12497958; doi:10.1093/cid/ciaf339)
Supplement: ciaf339_Supplementary_Data [file ciaf339_supplementary_data.zip › Manaus_Parra_Suplemental materials_revised.docx]

**Supplementary Material**

**Title**: Incidence and risk factors of SARS-CoV-2 infection among vaccinated healthcare workers during emergence of SARS-CoV-2 Gamma variant in the Amazon Region, Brazil 2021

**Authors:** Gemma Parra^1‡^, Fernanda C. Lessa^1,2‡^, Evelyn Campelo^3^, Tatyana C. Amorim Ramos^3^ ,Antonio Vieira^2^, Pritesh Lalwani^4,5^, Lucia I. Nichiata^7^, Luciana Silva-Flannery^2^, Charlene Siza^1,2^, Kassia Janara Veras Lima^7^, Aida Cristina Tapajos^8^, Ariana Vieira^8^, Mateusz Plucinski^2^, Barbara J. Marston^2^, Juliette Morgan^9^, Roberto Esteves^9^, Cristiano Fernandes da Costa ^10^, Felipe G. Naveca^11^, Maria Clara Padoveze^6^ for the Brazil Healthcare Personnel COVID-19 team

^1^ Division of Healthcare Quality Promotion, Centers for Disease Control and Prevention, Atlanta, USA

^2^ COVID‐19 Response ‐ International Task Force, Centers for Diseases Control and Prevention (CDC), Atlanta, Georgia, USA

^3^ Fundação de Vigilância em Saúde do Estado do Amazonas, Manaus, AM, Brazil

^4^ Instituto Leônidas e Maria Deane (ILMD), Fiocruz Amazônia, Manaus, Amazonas, Brazil

^5^ Laboratory of infectious diseases and immunology, ILMD/Fiocruz Amazônia and PPGIBA/ICB-UFAM, Manaus, Brazil

^6^ Universidade de São Paulo, Escola de Enfermagem, São Paulo, SP, Brazil

^7^Hospital Pronto Socorro 28 de Agosto, Manaus, AM, Brazil

^8^ Hospital Pronto Socorro Platão Araújo, Manaus, AM, Brazil

^9^ South America Regional Office, Centers for Diseases Control and Prevention (CDC), Brasília, DF, Brazil

^10^ Conselhos de Secretários Municipais de Saúde do Amazonas, Manaus, AM, Brazil

^11^ Laboratório de Ecologia

**Table S1: Classification of aerosol generating procedures in healthcare settings**

| **Risk group for potential aerosol generation among selected procedures*** |
| --- |
| **High** |
| Tracheal Intubation |
| Noninvasive ventilation |
| Cardiopulmonary resuscitation |
| Manual ventilation before intubation |
| Bronchoscopy |
| Sputum induction. |
| **Medium** |
| Nebulization |
| High-flow O2 |
| **Low** |
| Airway suctioning |
| Extubation |
| Respiratory physiotherapy |
| Bronchoalveolar lavage |

*Adapted from World Health Organization. https://www.who.int/publications/i/item/WHO-2019-nCoV-IPC-2021.1.

Table S2. Aerosol generating procedures performed by healthcare workers grouped by risk level for 4 weeks follow-up, March–May 2021.

| **Procedure Risk Level** | **Week 1**  N = 771 | | **Week 2**  N = 771 | | **Week 3**  N = 771 | | **Overall**  N = 2313 | |
| --- | --- | --- | --- | --- | --- | --- | --- | --- |
|  | **n** | **%** | **n** | **%** | **n** | **%** | **n** | **%** |
| High Exposure Risk | 160 | 21 | 137 | 18 | 129 | 17 | 426 | 18 |
| Medium Exposure Risk | 73 | 9 | 57 | 7 | 44 | 6 | 174 | 8 |
| Low Exposure Risk | 39 | 5 | 35 | 5 | 23 | 3 | 97 | 4 |
| No exposure | 499 | 65 | 542 | 70 | 575 | 75 | 1616 | 70 |

Figure S1. Personal Protective Equipment use* among healthcare workers (HCW) that reported caring for COVID-19 patients during the study period.
