# Supplementary figures and images for: Incidence of and Risk Factors for SARS-CoV-2 Infection Among Vaccinated Healthcare Workers During Emergence of SARS-CoV-2 Gamma Variant in the Amazon Region, Brazil, 2021
Source: Clin Infect Dis. 2025 Jul 30;81(3):451–8. doi: 10.1093/cid/ciaf339 (PMC12497958; doi:10.1093/cid/ciaf339)

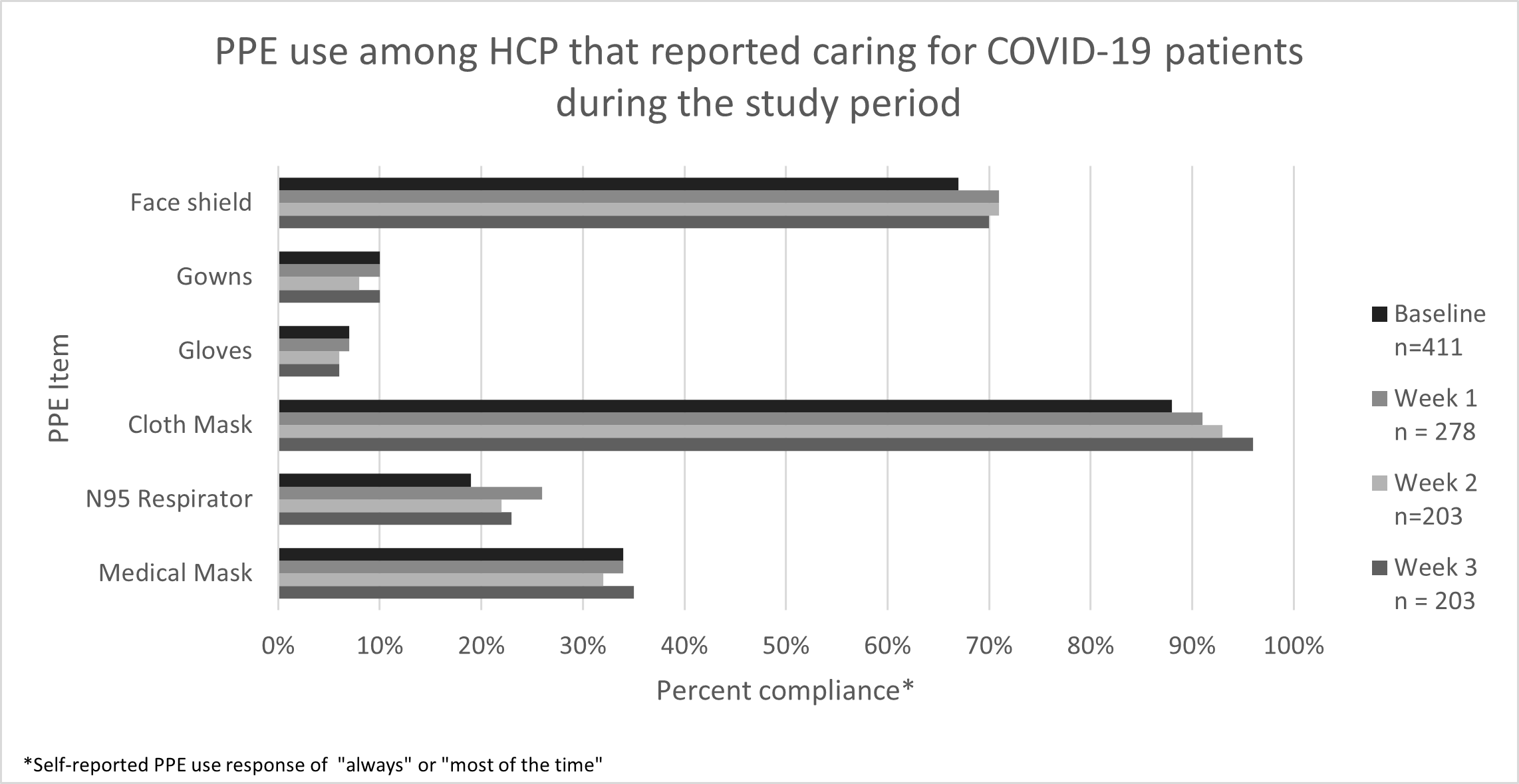

Supplement: ciaf339_Supplementary_Data [file ciaf339_supplementary_data.zip › S Fig 1 Parra.tif]
